# Severe acute syphilitic posterior placoid chorioretinitis with complete spontaneous resolution: The natural course

**DOI:** 10.3205/oc000039

**Published:** 2016-02-16

**Authors:** Mónica Franco, Vanda Nogueira

**Affiliations:** 1 Instituto de Oftalmologia Dr. Gama Pinto, Lisboa, Portugal

**Keywords:** syphilis, acute syphilitic posterior placoid chorioretinitis, immune response

## Abstract

**Purpose:** We report on a case of unilateral acute syphilitic posterior placoid chorioretinitis (ASPPC) with spontaneous resolution of the lesions, and discuss the role of an altered versus adequate immune response as the major pathogenic factor.

**Methods:** We describe a case of acute loss of visual acuity (VA) in the left eye (LE) in a 55-year-old healthy man.

**Results:** The patient presented with VA of 20/20 in the right eye (RE) and hand movements in the LE. Fundoscopy revealed a large yellowish placoid macular lesion with subretinal fluid in the LE, with no abnormalities detected in the RE. Fluorescein angiography showed early hypofluorescence with late staining in the affected area. The clinical findings progressed fast during the first week, with extension of the initial lesion outside the temporal retinal vascular arcades and the appearance of new lesions in the same eye. The patient abandoned the clinic for two weeks with no treatment. When observed again, VA of the LE had recovered to 20/20 and the lesions had completely resolved. Venereal disease research laboratory (VDRL) and fluorescent treponemal antibody absorption (FTA-ABS) tests results were positive and HIV antibody test titers negative. The diagnosis of ASPPC in the left eye was made. The patient accepted treatment with penicillin G only 45 days after the initial presentation. AV remained stable at 20/20 both eyes and no relapses of the lesions were observed during this period without therapy. The patient was followed for 3 months after treatment. He remained asymptomatic and the ophthalmic examination was unremarkable.

**Conclusions:** The pathogenesis of ASPPC is still not understood. Our case showed a sequential pattern of the chorioretinal lesions, with initial aggravation and complete posterior spontaneous resolution, showing the natural course of the disease. These findings suggest the presence of an adequate ocular immune response in patients with ASPPC, not supporting the initially proposed hypothesis of the importance of a modified immune response as the major pathogenic factor.

## Introduction

Syphilis is a sexually transmitted, systemic infection caused by the spirochete bacterium *Treponema* *pallidum* [1], with increasing incidence in the USA and Europe [2], [3], [4]. Ocular involvement may be silent or present as anterior uveitis, choroiditis, interstitial keratitis, retinal vasculitis, retinitis, optic neuritis, dacryoadenitis, or scleritis [5], [6], [7]. Acute syphilitic posterior placoid chorioretinitis (ASPPC) is a rare manifestation of syphilis, characterized by large, yellow-white geographic lesions involving the macula at the level of the outer retina/retinal pigment epithelium (RPE) [8]. It can present in immunocompetent and immunodepressed patients and the pathogenesis still remains unknown. Penicillin is the mainstay of treatment and is usually given early after serologic diagnosis of syphilis, so the natural course of the ocular lesions in not known. We present an untreated patient with complete spontaneous resolution of ASPPC.

## Purpose

To report on a case of unilateral acute syphilitic posterior placoid chorioretinitis in an immunocompetent patient with spontaneous resolution of the lesions and discuss the role of an altered versus adequate immune response as the major pathogenic factor.

## Case report

**Figure 1:**
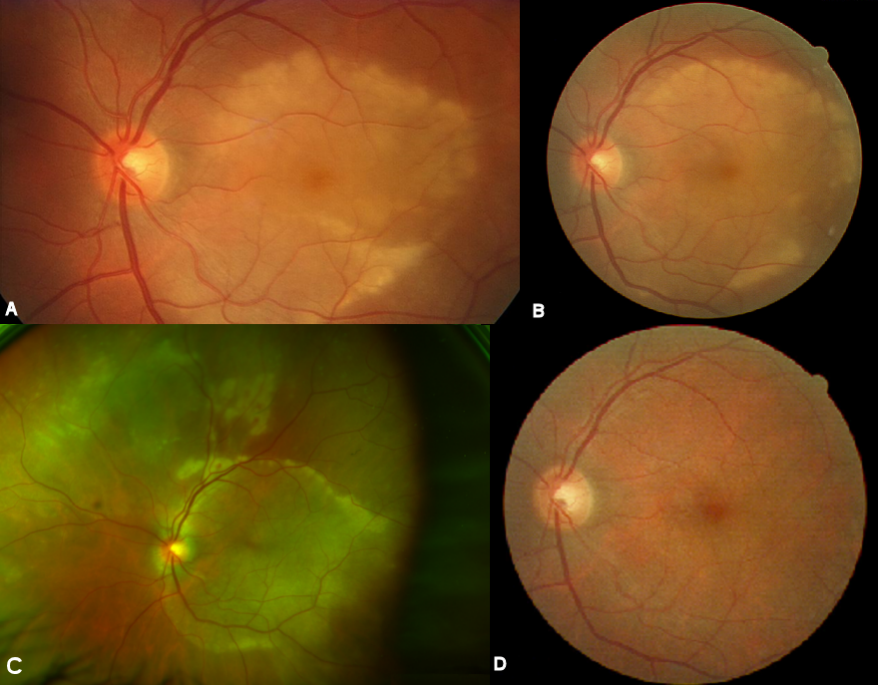
A) Colour photograph of the left eye shows a large yellow macular placoid lesion with a curvilinear edge. B) Colour photograph of the left eye, three days after the initial presentation, the placoid lesion had extended. C) Colour photograph of the left eye, one week after the initial presentation, placoid lesion had increased outside the temporal retinal vascular arcades and there are new multiple yellow lesions and hemorrhages in the retinal superior nasal area. D) Colour photograph of the left eye three weeks after presentation and before treatment shows the lesion’s complete resolution.

**Figure 2:**
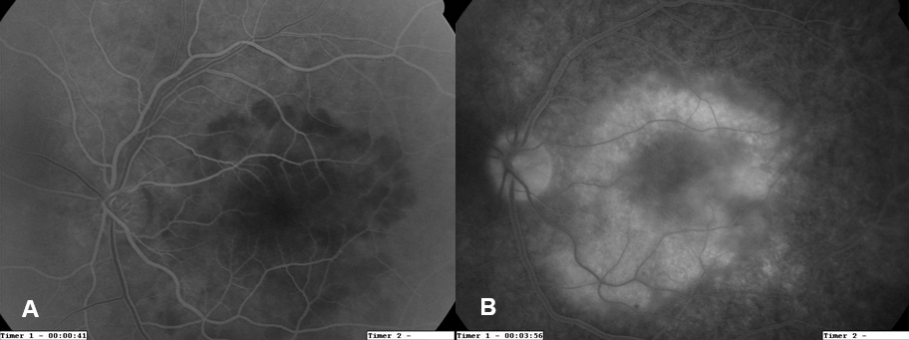
A) The early phase FA – corresponding to Figure 1A – shows hypofluorescence in the affected area. B) Late-phase FA shows progressive staining in the area of the lesion.

**Figure 3:**
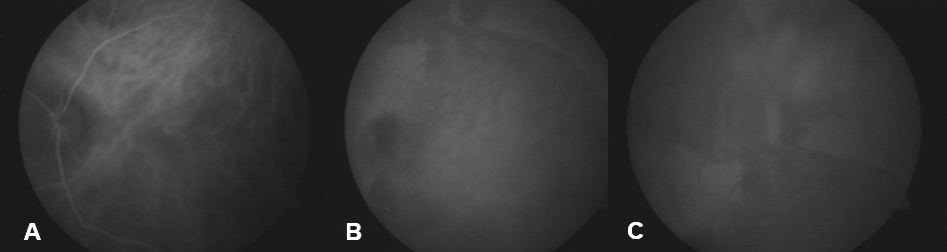
The ICGA – corresponding to Figure 1C – shows persistent leakage of the choriocapillaris and hypofluorescence areas in the early (A) and late phases (B, C).

A 55-year-old man presented with acute visual loss in the left eye (LE). The patient denied other ocular or systemic symptoms. He was not taking any medications and his medical, social and family history was unremarkable. Visual acuity (VA) was 20/20 in the right eye (RE) and hand movements in the LE. Slit-lamp examination findings of the anterior segment were normal in both eyes (OU) and intra-ocular pressure was 12 mmHg bilaterally. Dilated fundoscopy of the LE revealed a few vitreous cells and a large yellow macular placoid lesion with a curvilinear edge (Figure 1A (Fig. 1)). Fluorescein angiography (FA) showed early hypofluorescence with late staining in the affected area (Figure 2 (Fig. 2)). Optical coherence tomography (OCT) in the LE demonstrated subretinal fluid overlying the macular lesion (Figure 3 (Fig. 3)). No abnormalities were detected in the RE. Screening blood tests, including syphilis serology, were requested and the patient was monitored without treatment.

**Figure 4:**
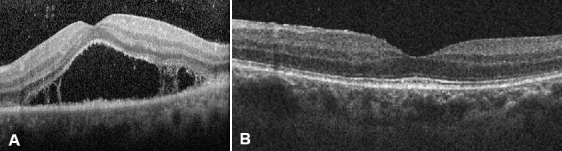
A) OCT of the left eye at presentation reveals subretinal fluid overlying the macular lesion. B) OCT image three weeks after presentation and before treatment demonstrates complete anatomical resolution.

Three days after the placoid lesion had extended (Figure 1B (Fig. 1)). The patient did not follow our recommendation for serologic work-up and the tests were ordered again. One week after the initial presentation, VA remained stable but the placoid lesion had increased outside the temporal retinal vascular arcades (Figure 1C (Fig. 1)). New multiple yellow lesions and hemorrhages were observed in the retinal superior nasal area. Indocyanine green angiography (ICGA) revealed persistent leakage of the choriocapillaris and hypofluorescence areas in the early and late phases (Figure 4 (Fig. 4)). The patient continued not to follow our recommendation for the blood tests and they were ordered for the third time. He was informed again about his clinical situation and the importance of the work-up was underlined. 

The patient abandoned the clinic for two weeks and when observed again, VA had improved to 20/20 in the LE and the lesions showed complete spontaneous resolution (Figure 1D (Fig. 1)). RE examination remained normal. Finally, we had access to the serologic tests results that revealed a positive VDRL (1/128) and FTA-ABS. HIV titers were negative. All other tests were normal. The diagnosis of acute syphilitic posterior placoid chorioretinitis in the LE was made. The patient was referred to the Infectious Disease Department. He had no other syphilitic manifestations and no abnormalities were detected after computed tomography of the cranium and orbits. A lumbar puncture revealed a positive VDRL and CSF-TPHA in the cerebrospinal fluid (CSF). The patient accepted to start treatment only 45 days after the initial presentation. During this period no relapse of the ocular lesions occurred and no systemic manifestation was observed. The patient was treated with intravenous aqueous penicillin G (four million units given every 4 h for 2 weeks). Three months after treatment, VA was 20/20 and fundoscopy remained normal both eyes. Follow-up serologic testing revealed a decrease of VDRL to 1/32.

## Discussion

Acute syphilitic posterior placoid chorioretinitis is a variant of syphilitic chorioretinitis characterized by a solitary large placoid lesion localized in the macular or juxtapapillary areas, first described by de Souza et al. [9] and subsequently by Gass et al. [8], who coined the term. Thought to occur primarily in immunocompromised patients, was also described in immunocompetent individuals [10], [11], [12], [13]. Although the fundoscopic and angiographic features of ASPPC are quite distinctive and suggestive of primary inflammation at the level of the choriocapillaris-retinal pigment epithelium complex, the histopathology and pathogenesis of this entity remain unknown. 

The role of a modified response of the immune system to syphilis, acting as a modulator of the clinical features, has been proposed as the major pathogenic mechanism. Tran et al. [14] suggested that individuals co-infected with HIV seem to have more severe ocular syphilis. Erol and Topbas [15] and Song et al. [16] described cases of ASPPC after an intravitreal triamcinolone acetonide injection. Brito et al. [17] reported an increased level of anti-beta2 glycoprotein I antibody in a patient with ASPPC, an apolipoprotein that binds to cardiolipin. The authors suggested these antibodies could cause focal choroidal thrombosis and altered retinal pigment epithelium metabolism, impairing photoreceptor function. Zamani and Garfinkel [18] also underlined the importance of the immune status in modulating the clinical signs by reporting the cause-and-effect relationship between oral prednisolone and ASPPC. 

Nevertheless, Yoo et al. [11] questioned this relationship by reporting a case of bilateral ASPPC in which the antibiotics were started late in the course of the disease. Chorioretinitis presented at different stages in both eyes and, regardless of the treatment with prednisone, the same clinical course was observed: onset-aggravation-resolution. The authors considered this an atypical case but suggested ASPPC could have its own clinical course, independent of steroid use. 

Our immunocompetent patient presented with severe ASPCC. Since the diagnosis was delayed, no treatment was started. Over one week we observed deterioration of the clinical features with enlargement of the lesion outside the macular limits and to the nasal quadrant. After this period complete spontaneous recovery was noticed and the patient was free of any sign of inflammation for 2 months, until he accepted to receive antimicrobial therapy to treat the systemic disease. To our knowledge this is the first report of a non-treated patient with complete anatomical and functional resolution of ASPPC. This shows the natural course of the disease not yet reported, since patients usually receive prompt antimicrobial treatment after simple serologic investigation. Eandi et al. [10] recently reported a case series of sixteen patients, all of them treated. We found no reports of untreated patients. Yoo et al. [11] and Meira-Freitas [19] described two patients initially not treated that showed a sequential pattern similar to our case. Nevertheless, even if the inflammation showed signs of spontaneous improvement, it subsided completely only after antimicrobial treatment. In addiction, both patients received early systemic steroids, a confounding factor to the disease’s natural course. 

We believe that this sequential pattern with complete spontaneous resolution of inflammation reflects an adequate response of the immune system to this infection, being able to control it locally. The presence of spirochetes in the eye is probable, since they are wide disseminated in secondary syphilis and present, for example, in the mucocutaneous lesions that accompanies 44% of the patients with ASPPC [10]. So the ocular sequential pattern is probably equivalent to the one of mucocutaneous lesions, which disappear even if not treated. 

This way, our findings are not consistent with the suggestion that the clinical features of ASPPC are secondary to an altered immune response to syphilis. Supporting our hypothesis is the fact that ASPPC presents in immunocompetent and immunocompromised patients, there are no consistent differences regarding clinical or angiographic manifestations in HIV positive and negative patients [10] and the suggestion that corticosteroids started before antimicrobial therapy could have no effect on the clinical appearance [11]. Our case also shows that even if not treated, a good visual outcome is possible, contrary to the general belief after the description of ASPPC by Gass et al. [8]. 

By better understanding the response to infectious agents, we could learn about our immune system, his defense mechanisms and the pathophysiology of infectious diseases.

To prevent damage in the eye and to avoid other manifestations of a systemic disease frequently undiagnosed, adequate antimicrobial treatment should always be prescribed to patients with ASPPC. 

## Notes

### Competing interests

The authors declare that they have no competing interests and that no financial support was received for this submission.

### Patient’s consent

The authors obtained written consent from the patient for the publication of her anonymised clinical data.
